# Determination of the Attenuation Coefficients of Epoxy Resin with Carbopol Polymer as a Breast Phantom Material at Low Photon Energy Range

**DOI:** 10.3390/polym15122645

**Published:** 2023-06-11

**Authors:** Mohammad Marashdeh, Muthanna Abdulkarim

**Affiliations:** 1 Department of Physics, College of Sciences, Imam Mohammad Ibn Saud Islamic University (IMSIU), P.O. Box 90950, Riyadh 11623, Saudi Arabia; 2 Department of Pharmaceutical Sciences, College of Pharmacy, Alfaisal University, P.O. Box 50927, Riyadh 11533, Saudi Arabia; malbaldawi@alfaisal.edu

**Keywords:** epoxy resin, Carbopol 974p polymer, X-ray fluorescent (XRF), phantom, attenuation coefficients, CT number

## Abstract

Six different composites of epoxy resin and Carbopol 974p polymer were prepared based on Carbopol 974p polymer concentrations of 0%, 5%, 10%, 15%, 20%, and 25%. The linear and mass attenuation coefficients, Half Value Layer (HVL), and mean free path (MFP) of these composites were determined using single-beam photon transmission in the energy range between 16.65 keV and 25.21 keV. This was carried out by determining the attenuation of k_a1_ X-ray fluorescent (XRF) photons from niobium, molybdenum, palladium, silver, and tin targets. The results were compared with theoretical values of three types of breast material (Breast 1, Breast 2, Breast 3) and Perspex, which was calculated using a XCOM computer program. The results show that there were no significant differences in the attenuation coefficient values after the consequent Carbopol additions. Moreover, it was found that the mass attenuation coefficients of all tested composites were close to those of Perspex and the values for Breast 3. The HVL and MFP results showed that the E25 sample is closer to the results of the Perspex material with differences of (0.53–1.15%) and (0.51–1.20%), respectively. In addition, the densities of the fabricated samples were in the range of 1.102–1.170 g/cm^3^, which is in the range of human breast density. A computed tomography (CT) scanner was used to investigate the CT number values for the fabricated samples. The CT numbers of all samples were in the range of human breast tissue (24.53–40.28 HU). Based on these findings, the fabricated epoxy–Carbopol polymer is a good candidate for use as a breast phantom material.

## 1. Introduction

Breast cancer is a major, global, unmet clinical condition which affects millions of people of all ages. Mammography is the premier method for breast cancer screening, detection, and assessment. Breast phantoms are generally used for mammography equipment testing and optimization, the teaching of radiologic professionals, and improvements to diagnostic quality control. The use of breast phantoms for the standardization of mammographic imaging techniques has significantly increased due to its high capacity to provide accurate and consistent mammographic interpretation [1].

There has been extensive research undertaken to develop breast phantom materials with similar radiologic features and tissue density to human breast tissue for accurate interpretation of mammograms [2,3]. Argo et al. [4] made a set of breast tissue equivalent phantoms (BRTEs) to mimic mammary glandular and fat tissue in terms of how they behave and how they absorb radiation. The BRTE phantom was made using epoxy resin, a hardener, magnesium oxide, polyethylene powder, and phenolic microspheres. Accordingly, Almeida et al. [5] prepared a series of breast tissue equivalent (BTE) phantoms using the same raw materials in the BRTE phantom made by Argo et al. and then tested them for mammographic dosimetry [4]. Almeida et al. [5] found that the linear attenuation coefficient for adipose and glandular BTE materials agreed well with the referenced breast phantom. Additionally, it was suggested that BTE phantoms be used to replace polymethyl methacrylate (PMMA) phantoms when measuring the average glandular dose and improving the quality of mammograms.

When epoxy resin is mixed with polymers such as Carbopol polymer, we can obtain a composite material with better mechanical and physical properties [6]. Polyacrylic acid is a common adjuvant chemical used in real-world cosmetology and pharmacology. It is also known as carboxypolymethylene, carboxyvinyl polymer, or carbomer. In order to condense liquids, stabilize suspensions, and produce tablets and capsules, Carbopol is employed in cosmetics and pharmacy industries [7]. Products containing the symbol P, including Carbopol 974P, are allowed for oral administration and mucosal contact. Carbopol 974P is frequently used in a variety of home, personal care, and pharmaceutical goods as a thickening agent, suspending agent, and viscosity enhancer. The polymer can also be utilized as a binder for tablets and other solid dosage forms, as well as a stabilizer for emulsions and dispersions [8]. In addition, Carbopol polymer is a hydrogel with high water content and a gel-like consistency. It is often used in soft and moldable materials to look similar to real breast tissue. Carbopol 974p is a highly crosslinked polyacrylic acid polymer in which the acrylic acid monomers are polymerized in ethyl acetate and crosslinked with allyl pentaerythritol. This polymer is highly used in various drug delivery applications, such as topical oral, nasal, ophthalmic, and vaginal drug delivery [9]. The reason behind the wide use of Carbopol 974p in pharmaceutical applications is due to its properties of being biodegradable, bio-adhesive, non-irritant, not absorbed into the body, and less expensive [10]. Moreover, Carbopol 974p is very safe for medical applications, with no evidence of any sort of toxicity (such as genotoxicity or liver toxicity) and with an acceptable daily limit of 1000 to 30,000 mg/kg [11]. In addition, studying the potential use of epoxy resin with Carbopol polymer as a phantom material at low energies has not been studied previously.

In terms of physical properties, this polymer is highly mucoadhesive, which makes it a very good choice for being used in topical applications to mucous membranes as it increases the contact time of the drug with the mucosal membrane [12].

In many different applications, such as radiation protection, medical imaging, and industrial radiography, radiation attenuation is an important characteristic for both shielding and phantom materials. Attenuation refers to the ability of a material to reduce the intensity of radiation as it passes through it [13]. In the case of shielding materials, attenuation is crucial to prevent radiation from penetrating the material and reaching the sensitive equipment or personnel behind it. The higher the attenuation of a material, the better it is at shielding against radiation. There are many studies examining the possibility of using new materials that have good physical properties and radiation protection [14,15,16,17]. In the case of phantom materials, which are used to simulate the behavior of human tissue in medical imaging and radiation therapy, attenuation is important in mimicking the absorption and scattering of radiation that occurs in the human body. Phantom materials with similar attenuation properties to human tissue can provide more accurate and realistic results in imaging and treatment planning [13]. Therefore, understanding and controlling the attenuation properties of materials is critical in many fields where radiation is involved.

The aim of this study is to investigate a new epoxy–polymer phantom material with a focus on its applicability in mammography applications. Therefore, in this study, the XRF method was used to measure the linear and mass attenuation coefficients of Araldite GY-6010 epoxy resin with Carbopol 974p polymer samples in the photon energy range of 16.65–25.21 keV. These results were compared with the theoretical values for three types of breast material for young age (Breast 1), middle age (Breast 2), and old age (Breast 3) groups and polymethyl methacrylate (PMMA), known as Perspex, with results calculated from the XCOM program [18]. Moreover, the HVL and MFP values of fabricated samples were calculated and compared with those of Perspex at the XRF energy range of 16.65–25.21 keV. In addition, the CT number value in the fabricated samples was determined.

## 2. Materials and Methods

### 2.1. Samples Preparation

Different ratios of epoxy resin (Araldite-GY6010, supplied by JANA, KSA), Carbopol 974p polymer powder, and a polyether amine hardener (Jeffamine-T403, Huntsman Chemical, The Woodlands, TX, USA) were used to obtain composites at different ratios. The epoxy sample composites were fabricated through manual mixing of epoxy resin with different amounts of Carbopol polymer (0%, 5%, 10%, 15%, 20%, and 25%). To ensure good homogeneity in the composites, all the samples were mixed manually for an additional 15 min to prevent the introduction of air bubbles in the fabricated sample, which can affect the homogeneity and density of samples. After that, each mixture was poured into an approximately 1 cm deep rectangular mold made of Teflon, and the full cure time of the mixer was 48 h at 23 °C, which is the amount of time required for the epoxy to fully harden. As the maximum thickness of the fabricated samples was 1 cm, a longer time was needed to ensure that the full cure time was achieved. Finally, samples were taken out of the molds, and the edges of the samples were smoothed to the required dimensions. For each type of sample, rectangular cuboids with three different thicknesses (1 cm, 0.6 cm, and 0.4 cm) were fabricated. The thicknesses were determined based on a target density of 1.00 g/cm^3^. In addition, the density of the cuboid samples was simply calculated by dividing the sample mass (g) by the sample volume (cm^3^) (Table 1).

### 2.2. Linear and Mass Attenuation Coefficient Measurements

**Figure 1:**
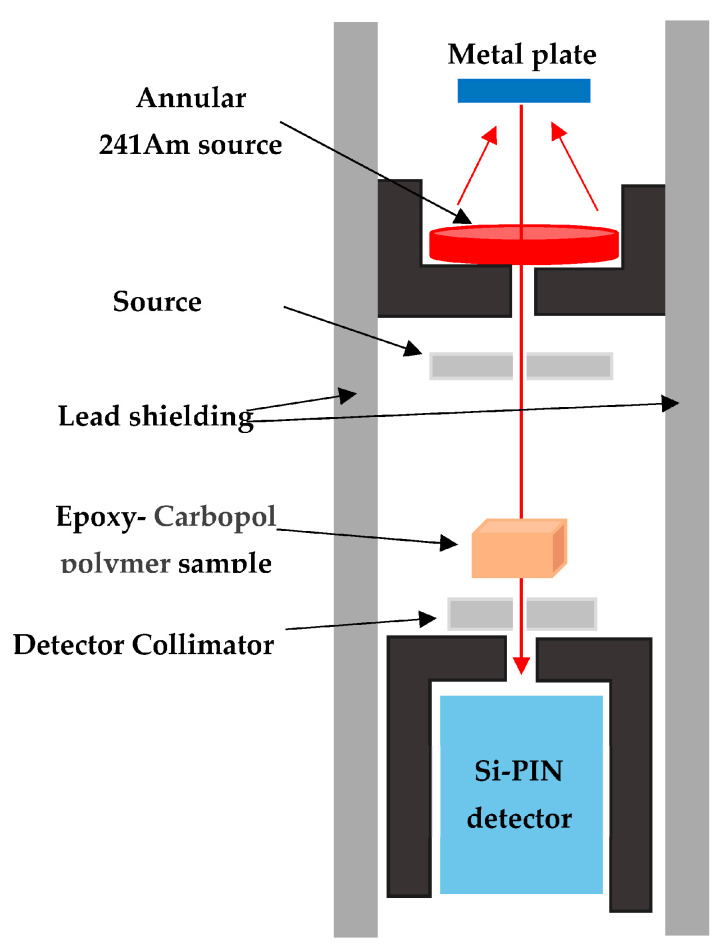
Schematic showing the experiment’s setup.

Linear and mass attenuation coefficients were tested using an X-ray fluorescent (XRF) system equipped with pure metal plates comprising niobium (Nb), molybdenum (Mo), palladium (Pd), silver (Ag), and tin (Sn) with photon energies of 16.65, 17.44, 21.16, 22.18, and 25.21 keV, respectively. Samples were irradiated with XRF energies from an ^241^Am radioactive annular source with an activity of 3.7 GBq. Table 2 summarizes the characteristics of the metal plates utilized in this experiment. The experimental setup is depicted in Figure 1 and was based on the same principles and methodology that our group previously described [19,20,21]. XRF energy intensities were detected using a Si-PIN photodiode XR-100 CR detector with a thickness of 300 μm and an active area of 7 mm^2^. To ensure the accuracy of the results, the samples were irradiated for 2000 s. Similarly, to block any scattering or background radiation, a collimator 0.5 cm in diameter was equipped in the Si-PIN detector. The distances between the pure metal plate, sample, and detector were determined to be 17 cm and 14 cm, respectively, which was achieved by conducting a final adjustment procedure using a gamma-ray source and observing the beam at the detector collimator. Beam alignment was assessed multiple times by varying the distances between the metal plate and the sample and the sample and the detector, confirming conformity with all parameters of an XRF system; fabricated samples (E0–E25) with thicknesses of 1.0, 0.6, and 0.4 cm were utilized.

The findings of the spectra of metal plates with and without samples were used to identify the region of interest for k_α1_ peak energies. The peak’s corresponding channel number was noted. For the attenuated and unattenuated X-ray fluorescence spectra of the metal plate targets, the net area value was recorded as *I* and *I_o_*, respectively. The amount from the background counts is the net area. 

The mass attenuation coefficient *μ/ρ* (cm^2^/g) can be obtained when the linear attenuation coefficient is divided by the density of the sample according to Beer–Lambert’s law, which is given by

(1) $$\mu /\rho =\frac{1}{\rho x}\ln\left(\frac{I_{o}}{I}\right)$$

where *I_o_* denotes photon intensity without attenuation, *I* is photon intensity after attenuation, and *ρx* is area density, also known as mass thickness.

The Half Value Layer (*HVL*), which is defined as the thickness needed to reduce the radiation intensity in half, is as follows:

(2) $$HVL=\frac{ln2}{\mu }$$

The thickness that a photon travels before interacting is referred to as the mean free path. The inverse expression of the sample materials’ linear absorption coefficient can be found using

(3) $$MFP=\frac{1}{\mu }$$

### 2.3. X-ray Computed Tomography (CT) Scanner

The CT number was investigated using an X-ray computed tomography scanner (Somaton Sensation Open, Siemens, Munich, Germany). The machine’s maximum voltage and current settings were 120 kVp and 33 mA, respectively, which generates an image pixel value known as the CT number. Tungsten was employed as the X-ray target. The CT number is defined by the following equation:

(4) $$\mathrm{CT}\;\mathrm{number}=K\frac{\mu -\mu_{w}}{\mu_{w}}$$

where μ is the linear attenuation coefficient of the sample, μ_w_ is the linear attenuation coefficient of water, and K is a magnification contrast (=1000) [22]. The Hounsfield unit (HU) value, which is a density-related number, was 0 and −1000 for water and air, respectively.

**Figure 2:**
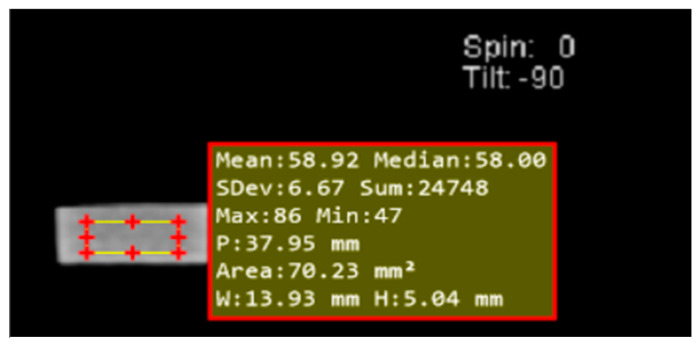
Example CT image of the cross-sectional area with the area of interest.

The fabricated samples were scanned with approximately 9 beams, each 3 mm in thickness. As shown in Figure 2, the region of interest was investigated using a 70 mm^2^ rectangle. To decrease the pixel averaging effect at the interface of fabricated samples and air at the edge area, the CT number values were not captured during the beginning and end scans. Other average CT number values of 7 or 8 were calculated.

## 3. Results and Discussion

### 3.1. Mass Attenuation Coefficient Measurements

**Figure 3:**
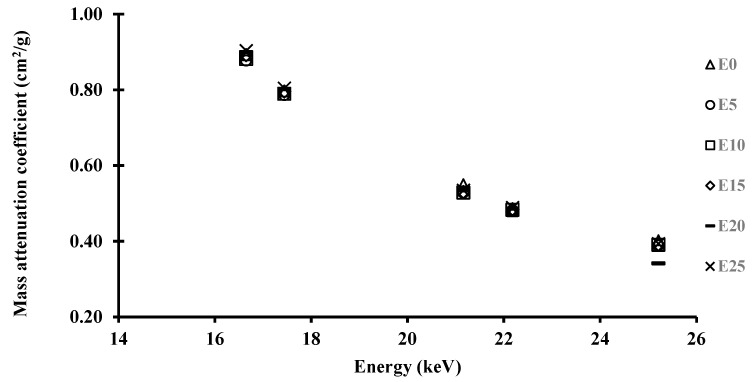
Mass attenuation coefficient of the epoxy resin–Carbopol 974p polymer samples at different k_a1_ XRF peak energies.

In this study, the linear attenuation coefficients (μ) of various epoxy resin composite samples were measured within the gamma energy range of 16.65–25.21 keV. The results revealed that μ somewhat increased with the addition of Carbopol 974p polymer. The presence of a Carbopol 974p polymer with a different atomic number (Z) in the composites, which allows photons to be available for gamma-ray interaction, might explain this observation. Table 3 also shows the mass attenuation coefficients (*μ/ρ*) of the epoxy samples made from niobium, molybdenum, palladium, silver, and tin in the photon energy range of 16.65–25.21 keV using different X-ray fluorescent beam (XRF) energies, with errors of 0.042% to 0.406%. The net counts under the k_a1_ peak were used to calculate the strengths of the incident and transmitted beams. Figure 3 depicts the relationship between the observed mass attenuation coefficients and the percentage of Carbopol 974p polymer additive. While the *μ/ρ* values of composite samples fall as energy increases, these *μ/ρ* values rise as sample thickness increases.

As can be seen in Figure 3, the distribution of *μ/ρ* values fell exponentially as gamma-ray energy increased. These findings clearly suggest that the contrast of the mass attenuation coefficient with gamma rays varies with energy, which is attributable to the many photon–material interaction processes. Photoelectric absorption was the major interaction between photons and epoxy resin composite samples in this energy range of 16.59 keV to 25.26 keV.

**Figure 4:**
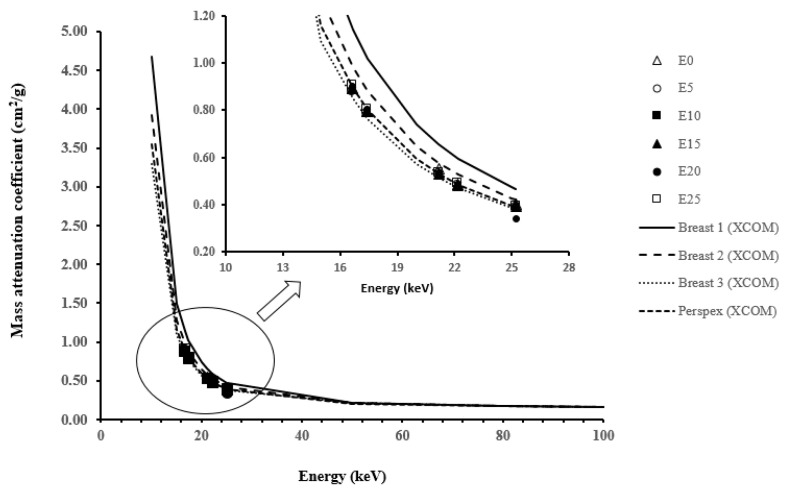
Mass attenuation coefficients at different energies for the E0, E5, E10, E15, E20, and E25 samples compared with calculated XCOM values for Breast 1, Breast 2, Breast 3, and Perspex.

Figure 4 shows the *μ/ρ* values of the fabricated samples in comparison to the values for Breast 1, Breast 2, Breast 3, and Perspex, which were established by XCOM based on the proposed elemental composition described by Constantinou [23]. It can be seen that there were no significant differences in the attenuation coefficient values in response to consequent polymer additions. All samples’ mass attenuation coefficients were shown to be quite close to those of Perspex and Breast 3, as shown in the zoomed-in area in Figure 4.

The significance of incorporating a Carbopol 974P polymer into the Araldite GY-6010 epoxy resin, which is utilized as a phantom material in medical applications, is thus made clearly obvious. Furthermore, addition of Carbopol 974P polymer to Araldite GY-6010 epoxy resin can be accompanied by a variety of benefits depending on the particular application and desired qualities. The benefits of Carbopol 974P polymer addition lead to its is frequent use as a thickener and rheology modifier in a variety of industries, including personal care, drugs, and coatings. It may increase the epoxy resin’s viscosity and stability, as well as its adhesion and flow characteristics, and it can provide more control over the curing process. It can also improve the epoxy resin’s impact resistance, toughness, and mechanical strength [24].

The percentage of deviation in the mass attenuation coefficients of samples E0, E5, E10, E15, E20, and E25 from the calculated values of Breast 1, Breast 2, Breast 3, and Perspex is shown in Table 4. It was found that there was a noticeable deviation in comparison with Breast 1 (percentage difference of about 15.462–26.714%), while there was no significant deviation between the epoxy samples and the Breast 2, Breast 3, and Perspex samples. Most of the deviations in the fabricated epoxy samples are in the range of 10.736%, −7.454%, and −3.866%, respectively.

Even though Carbopol 974P and epoxy resin have different physical and chemical characteristics, it is still possible to combine the two to create a composite material that has the qualities needed for a breast phantom. The XRF attenuation coefficient of the fabricated sample is similar to that of human breast tissue. The abovementioned results show the suitability of using Carbopol 974P as a component in a composite of breast phantom material in breast imaging applications.

### 3.2. Half Value Layer (HVL) and Mean Free Path (MFP)

The aim of mammography imaging is to find breast cancer at an early stage while exposing the patient to the least amount of radiation possible. HVL and MFP measurements can assist in figuring out the ideal thickness and make-up of the phantom material so that appropriate image quality may be produced while exposing the patient to the lowest dose of radiation possible. The phantom material’s HVL and MFP measurements can help ensure that it effectively mimics the attenuation characteristics of breast tissue, which is essential to an accurate diagnosis. In this study, the results of the HVL and MFP measurements of the fabricated samples were obtained at a range of photon energies (16.65–25.21 keV) and compared with the calculated results for Perspex, as shown in Table 5. The results show that all epoxy–Carbopol polymer values are higher than the Perspex values, which represents the depth of X-ray penetration into the material that was used to determine the accuracy of the radiation dose received by the patient [25]. In addition, the results showed that sample E25 was closer to the results of the Perspex material, with a difference percentage of (0.53–1.15%) and (0.51–1.20%) for the HVL and MFP results, respectively, within the photon energies of 16.65–25.21 keV. Thus, the importance of using this fabricated material appears in terms of application as a phantom material, as it has the possibility of being developed through the addition of polymer materials, which can develop its physical and mechanical properties and the ease of fabricating it in the form intended so that it may be applied in various fields of radiography and associated applications.

### 3.3. CT Number Measurement

The X-ray attenuation coefficient of a material, in relation to its density, is expressed by the CT number, which is utilized in medical imaging. The CT number of an object is determined by contrasting it with water, which has a CT value of zero. Table 6 lists the average, maximum, minimum, and standard deviation values of the CT number value for the fabricated samples (E0, E5, E10, E15, E20, and E25). It is important to note that every CT value recorded for sample E0 through E25 falls within the average range of CT values found in breast tissue, which is normally in the range of +5 to +50 Hounsfield units (HU) [26]. In addition to the CT number, additional characteristics, such as density and stability over time, should be considered when evaluating a material’s viability as a breast phantom. In addition, it can be seen that the values of the CT number increase slightly with subsequent increases in the percentage of Carbopol 974P polymer but remain within the appropriate range to be considered concerning breast tissue results. In addition, the standard deviation value is generally lower in samples that do not contain the Carbopol 974P polymer (2.23), while it increases with the addition of Carbopol 974P polymer at different proportions (4.18–7.19) according to the distribution of the values of the CT number.

In addition to CT number, it is essential to consider a material’s density, in connection with X-ray attenuation, when determining its viability as a breast phantom material with the aimed density of 1.0 g/cm^3^, i.e., the density of breast tissue. The density of these fabricated samples was found to be between 1.102 and 1.170 g/cm^3^, which is comparable to the density of normal tissue, as shown in Table 5. Materials used as breast phantoms should have a density that is within the same range as breast tissue in order to accurately replicate the properties of breast tissue. The epoxy–polymer material has the potential to be used as a phantom material in medical and diagnostic radiological applications, and the results concerning CT number and density corroborate this.

## 4. Conclusions

In this study, the *μ* and *μ/ρ* values of six different epoxy resin composites with different amounts of Carbopol 974p polymer (0%, 5%, 10%, 15%, 20%, and 25%) were measured. The results were contrasted with theoretical estimates of three different breast materials (Breast 1, Breast 2, and Breast 3) and Perspex, which were produced by an XCOM computer program. This study demonstrates that the μ and *μ*/*ρ* values did not change significantly with changes to the ratio of Carbopol polymer. Additionally, it was discovered that the mass attenuation coefficient of the epoxy–Carbopol polymer was quite similar to the values of Breast 3 and Perspex. The percentage of deviation in the mass attenuation coefficients of all samples from the calculated values of Breast 3 and Perspex was −7.454% and −3.866%, respectively. The epoxy–Carbopol polymer samples also showed HVL and MFP values that are close to those of Perspex (a standard breast phantom material), especially sample E25; this can be a positive development for mammography applications. Additionally, the density of the fabricated samples was measured to be between 1.102 and 1.170 g/cm^3^, which is within the range of the density of a human breast. All of the CT number results for epoxy–Carbopol polymer samples fell within the range obtained for human breast tissue, which is between 24.53 and 40.28 HU. Consequently, the created epoxy–Carbopol polymer composite is a very good candidate for use as a breast phantom material.

## Figures and Tables

**Table 1:** Formulations of epoxy resin samples with different additives.

Samples	Concentration (wt.%)			Measured Density (g/cm^3^)
	Araldite-GY6010	Jeffamine-T403	Carbopol 974p Polymer	
E0	75.00	25.00	0.00	1.102
E5	71.25	23.75	5.00	1.110
E10	67.50	22.5	10.00	1.130
E15	63.75	21.25	15.00	1.150
E20	60.00	20.00	20.00	1.160
E25	56.25	18.75	25.00	1.170

**Table 2:** The parameters of metal plate materials, kα1 fluorescence energies in keV, and intensities in counts s^−1^.

Element	Atomic No. (Z)	Weight (g)	Thickness (mm)	Purity (%)	K_α1_ (keV)	I_o_ (Counts s^−1^)
Niobium	41	2.25	0.14	99.80	16.65	2616
Molybdenum	42	2.06	0.11	99.90	17.44	2639
Palladium	46	3.00	0.10	99.90	21.16	3169
Silver	47	13.20	2.00	99.99	22.18	3023
Tin	50	3.80	0.28	99.99	25.21	3217

**Table 3:** The parameters of metal plate materials, kα1 fluorescence energies in keV, and intensities in counts s^−1^.

	Nb			Mo			Pd			Ag			Sn		
	16.65 KeV			17.44 KeV			21.16 KeV			22.18 KeV			25.21 KeV		
Sample	μ cm^−1^	*μ*/*ρ* cm^2^/g	±σ (*μ*/*ρ*)	μ cm^−1^	*μ*/*ρ* cm^2^/g	±σ (*μ*/*ρ*)	μ cm^−1^	*μ*/*ρ* cm^2^/g	±σ (*μ*/*ρ*)	μ cm^−1^	*μ*/*ρ* cm^2^/g	±σ (*μ*/*ρ*)	μ cm^−1^	*μ*/*ρ* cm^2^/g	±σ (*μ*/*ρ*)
E0	0.982	0.891	0.127	0.877	0.796	0.050	0.607	0.551	0.068	0.529	0.480	0.052	0.444	0.403	0.049
E5	0.974	0.877	0.106	0.873	0.786	0.085	0.587	0.529	0.117	0.534	0.481	0.095	0.432	0.389	0.046
E10	0.996	0.881	0.110	0.892	0.790	0.075	0.597	0.528	0.120	0.546	0.483	0.110	0.441	0.391	0.081
E15	1.026	0.892	0.101	0.915	0.796	0.075	0.610	0.530	0.090	0.557	0.485	0.111	0.450	0.391	0.042
E20	1.043	0.899	0.099	0.930	0.802	0.062	0.620	0.534	0.065	0.564	0.486	0.061	0.396	0.341	0.070
E25	1.057	0.904	0.087	0.941	0.804	0.055	0.626	0.535	0.078	0.572	0.489	0.045	0.461	0.394	0.052

**Table 4:** The percentage of deviation in the mass attenuation coefficients of fabricated epoxy samples from the calculated Breast 1, Breast 2, Breast 3, and Perspex values (XCOM).

Percentage of Deviation from Breast 1						
ENERGY	E0	E5	E10	E15	E20	E25
16.65	21.884	23.113	22.749	21.845	21.166	20.800
17.44	21.740	22.703	22.348	21.763	21.188	20.910
21.16	15.756	19.122	19.155	18.890	18.292	18.187
22.18	19.120	18.958	18.551	18.305	17.978	17.571
25.21	13.544	16.532	16.166	16.069	15.789	15.462
Percentage of Deviation from Breast 2						
ENERGY	E0	E5	E10	E15	E20	E25
16.65	9.310	10.736	10.314	9.265	8.476	8.052
17.44	9.516	10.628	10.218	9.542	8.877	8.556
21.16	4.641	8.450	8.488	8.188	7.510	7.392
22.18	9.010	8.827	8.370	8.093	7.726	7.267
25.21	4.437	7.740	7.335	7.227	6.919	6.557
Percentage of Deviation from Breast 3						
ENERGY	E0	E5	E10	E15	E20	E25
16.65	−5.119	−3.466	−3.954	−5.171	−6.086	−6.577
17.44	−4.367	−3.083	−3.557	−4.336	−5.104	−5.474
21.16	−7.454	−3.161	−3.118	−3.456	−4.220	−4.354
22.18	−1.890	−2.095	−2.607	−2.917	−3.329	−3.842
25.21	−5.115	−1.481	−1.927	−2.045	−2.384	−2.783
Percentage of Deviation from Perspex						
ENERGY	E0	E5	E10	E15	E20	E25
16.65	0.146	1.716	1.251	0.096	−0.773	−1.240
17.44	0.550	1.773	1.321	0.579	−0.153	−0.505
21.16	−3.866	0.283	0.324	−0.003	−0.741	−0.870
22.18	1.154	0.956	0.459	0.158	−0.241	−0.739
25.21	−3.018	0.543	0.107	−0.009	−0.342	−0.733

**Table 5:** Results of the Half Value Layer (HVL) and mean free path (MFP) measurements of epoxy–Carbopol polymer fabricated samples and Perspex (XCOM) against photon energy.

Energy (keV)	E0		E5		E10		E15		E20		E25		Perspex (XCOM)	
	HVL (cm)	MFP (cm)	HVL (cm)	MFP (cm)	HVL (cm)	MFP (cm)	HVL (cm)	MFP (cm)	HVL (cm)	MFP (cm)	HVL (cm)	MFP (cm)	HVL (cm)	MFP (cm)
16.65	0.706	1.018	0.712	1.027	0.696	1.004	0.676	0.975	0.664	0.958	0.655	0.946	0.652	0.941
17.44	0.790	1.140	0.794	1.146	0.777	1.121	0.757	1.093	0.745	1.076	0.736	1.063	0.728	1.050
21.16	1.142	1.648	1.181	1.704	1.160	1.675	1.137	1.640	1.118	1.614	1.108	1.598	1.098	1.585
22.18	1.311	1.892	1.299	1.874	1.270	1.832	1.244	1.795	1.228	1.772	1.212	1.748	1.200	1.732
25.21	1.561	2.253	1.605	2.317	1.570	2.266	1.541	2.224	1.750	2.525	1.504	2.170	1.489	2.149

**Table 6:** The mean, maximum, minimum, and standard deviation values of the CT numbers for the 1 cm thick fabricated samples.

	CT Number				Calculated Density (g/cm^3^)
Sample	Mean	Maximum	Minimum	Standard Deviation	
E0	24.53	46.07	12.55	2.32	1.102
E5	27.02	49.61	11.83	7.19	1.110
E10	31.56	52.95	14.90	6.22	1.130
E15	37.66	55.39	12.48	5.32	1.150
E20	35.81	60.22	13.92	4.18	1.160
E25	40.28	65.45	14.67	4.77	1.170

## Data Availability

The data presented in this study are available on request from the corresponding author.
